# Effectiveness of an Internet-Based and Telephone-Assisted Training for Parents of 4-Year-Old Children With Disruptive Behavior: Implementation Research

**DOI:** 10.2196/27900

**Published:** 2022-04-04

**Authors:** Andre Sourander, Terja Ristkari, Marjo Kurki, Sonja Gilbert, Susanna Hinkka-Yli-Salomäki, Malin Kinnunen, Laura Pulkki-Råback, Patrick J McGrath

**Affiliations:** 1 Department of Child Psychiatry University of Turku Turku Finland; 2 Finland INVEST Research Flagship University of Turku Turku Finland; 3 Department of Child Psychiatry Turku University Hospital Turku Finland; 4 ITLA Children’s Foundation Helsinki Finland; 5 Department of Psychology and Logopedics Faculty of Medicine University of Helsinki Helsinki Finland; 6 Centre for Research in Family Health IWK Health Centre Halifax, NS Canada; 7 Faculty of Science Dalhousie University Halifax, NS Canada; 8 Strongest Families Institute Halifax, NS Canada; 9 Department of Psychiatry Dalhousie University Halifax, NS Canada

**Keywords:** parent training, early intervention, implementation, disruptive behavior, behavior problems, preschool children, internet-assisted, child mental health, mental health, behavior, intervention, children, parents

## Abstract

**Background:**

There is a lack of effectiveness studies when digital parent training programs are implemented in real-world practice. The efficacy of the internet-based and telephone-assisted Finnish Strongest Families Smart Website (SFSW) parent training intervention on the disruptive behavior of 4-year-old children was studied in a randomized controlled trial setting in Southwest Finland between 2011 and 2013. After that, the intervention was implemented nationwide in child health clinics from 2015 onwards.

**Objective:**

The main aim of this study was to compare the treatment characteristics and effectiveness of the SFSW parent training intervention between the families who received the intervention when it was implemented as a normal practice in child health clinics and the families who received the same intervention during the randomized controlled trial.

**Methods:**

The implementation group comprised 600 families who were recruited in the SFSW intervention between January 2015 and May 2017 in real-world implementation. The RCT intervention group comprised 232 families who were recruited between October 2011 and November 2013. The same demographic and child and parent measures were collected from both study groups and were compared using linear mixed-effect models for repeated measurements. The child psychopathology and functioning level were measured using the Child Behavior Checklist (CBCL) version 1.5-5 for preschool children, the Inventory of Callous-Unemotional Traits (ICU), and a modified version of the Barkley Home Situations Questionnaire. Parenting skills were measured using the 31-item Parenting Scale and the shorter 21-item Depression, Anxiety and Stress Scale (DASS-21). The estimated child and parent outcomes were adjusted for CBCL externalizing scores at baseline, maternal education, duration of the behavior problems, and paternal age. The baseline measurements of each outcome were used as covariates.

**Results:**

The implementation group was more likely to complete the intervention than the RCT intervention group (514/600, 85.7% vs 176/232, 75.9%, respectively; *P*<.001). There were no significant differences between the implementation and RCT intervention groups with regard to child measures, including CBCL externalizing score (–0.2, 95% CI –1.3 to 1.6; *P*=.83), total score (–0.7, 95% CI –3.0 to 4.5; *P*=.70), internalizing score (–0.3, 95% CI –1.0 to 1.6; *P*=.64), and ICU total score (–0.4, 95% Cl –1.9 to 1.2; *P*=.64). No significant difference was detected in the Parenting Scale total score (0.0, 95% Cl –0.1 to 0.1; *P*=.50), while DASS-21 total score differed nearly significantly (2.5, 95% Cl 0.0-5.1; *P*=.05), indicating better improvement in the implementation group.

**Conclusions:**

The internet-based and telephone-assisted SFSW parent training intervention was effectively implemented in real-world settings. These findings have implications for addressing the unmet needs of children with disruptive behavior problems. Our initiative could also provide a quick socially distanced solution for the considerable mental health impact of the COVID-19 pandemic.

**Trial Registration:**

ClinicalTrials.gov NCT01750996; https://clinicaltrials.gov/ct2/show/NCT01750996

**International Registered Report Identifier (IRRID):**

RR2-10.1186/1471-2458-13-985

## Introduction

### Background

There is mounting evidence from randomized controlled trials (RCTs) that parents can be trained to tackle and reduce children’s disruptive behavior and improve their parenting skills [1-3]. These findings are of upmost importance to public health professionals because children who exhibit disruptive behavior face increased risks of adult psychiatric disorders, substance use, crime, suicide, and other adversities [4,5]. Sufficiently strong evidence has been published on the efficacy of parent training to suggest that psychosocial services for children should include evidence-based parent training programs [6,7]. The need for services to tackle childhood disruptive behavior is enormous, but only a minority of families receive them [8]. There are challenges to implementing traditional face-to-face group-based parent training programs in real-world settings. One issue is the large number of barriers such as high cost, poor access, inconvenience, and low fidelity [3,9]. Another is keeping the content of the intervention consistent with the original evidence-based treatment [2].

Digitally assisted interventions are becoming more common, as they can overcome the barriers associated with conventional programs [3,9]. They are also likely to become increasingly popular, as child mental health services struggle to deal with the considerable increase in demand for their services as a result of the COVID-19 pandemic. This unprecedented global health emergency is expected to have major ongoing effects on child mental health owing to factors such as quarantine measures, social distancing, and school closures [10]. The pandemic started at a time when resources were already under pressure, and these are expected to be further affected by manpower shortages and a global recession that puts even greater pressure on health budgets. Digitally assisted interventions are cost-effective solutions that require fewer personnel and can reach geographically remote areas that would otherwise be outside of the reach of specialist services.

RCT studies have shown that remote and digitally assisted parent training programs have worked well in clinical settings [11,12]. We previously reported 12-month and 24-month follow-up studies of the first RCT on the Strongest Families Smart Website (SFSW). This RCT used a population-based sample and provided an internet-based parent training intervention with weekly telephone coaching [13-15]. The development of the SFSW intervention was based on the social learning and cognitive behavioral theories as well as positive parenting practices [16-18]. The target population was 4-year-old children who displayed high levels of disruptive behavior when they were screened during annual health checkups at child health clinics across Southwest Finland. The RCT showed that the children and parents who received the SFSW parent training program derived significant benefits from the initiative. The children displayed significant reductions in their disruptive behavior and other psychiatric symptom domains at their 24-month follow-up assessments. They also demonstrated the same improvements when they were compared with an education control group. The education control group received access to a static website that provided parents with information on how to tackle behavior problems and 1 phone call with a coach. Improved parenting skills were maintained in the intervention group at the 24-month follow-up assessment [14].

There has been growing interest in implementation research during the past 2 decades. Dissemination refers to how knowledge of new practices is actively and passively extended, and implementation refers to how new practices are incorporated into real-world environments. The term *implementation gap* is used to refer to the difference between our knowledge of *what works* and *how it works* [19,20]. Unfortunately, the strong effects that are observed in controlled RCT settings can weaken or become ambiguous when they are implemented in real-world settings [9]. Meta-analyses have shown that effective implementation has been associated with better outcomes, and the magnitude of the mean effect sizes was considerably higher when programs were carefully implemented and when fidelity was confirmed [21]. Successfully converting psychosocial interventions from experimental environments to real-world practice requires a solid framework and a structured implementation plan [22]. Research on evidence-based parent training programs after the RCT stage has often focused on examining the characteristics of an optimal implementation environment rather than maintaining the effectiveness of the intervention. We are not aware of any previous reports on the effectiveness of implementing digital interventions for disruptive behavior so that they can form part of the routine care that children below school age can receive.

### Objectives

This was the first study to report the effectiveness of the SFSW internet-based and telephone-assisted parent training program for preschool children when it was implemented in real-world settings. The intervention was put into practice after the population-based screening was used to identify children with disruptive behavior problems during routine visits to Finnish child health clinics at the age of 4 years. The primary aim was to report the changes in the children’s psychopathology and functioning level and any improvement in their families’ parenting skills. The children and their parents were followed up 6 months after the SFSW intervention was nationally implemented in Finnish primary care child health clinics. We compared the treatment characteristics and effectiveness between the families who received the SFSW intervention in these real-world settings from January 2015 to May 2017 and the families who received the intervention during the RCT from October 2011 to November 2013. Finally, we verified the findings by carrying out the following additional analyses. The first analysis excluded families who did not complete the parent training program. The second analysis excluded the Turku study site from the implementation study group because it was the only site that participated in both the RCT intervention and the implementation phases. In the third analysis, we compared the implementation and the RCT education control group. Our hypothesis was that the effectiveness of the SFSW intervention would be maintained if the protocol used in our previous RCT and the structured implementation plan were strictly adhered to.

## Methods

### Study Design

This study was a longitudinal comparison of 2 parallel groups. The implementation group comprised 600 families who received the SFSW internet-based and telephone-assisted parent training program in the real-world setting between January 2015 and May 2017. The implementation phase covered 95 child health clinics in 12 administrative regions across Finland. The RCT intervention group comprised 232 families who had been recruited by 42 child health clinics in 7 administrative regions in Southwest Finland between October 2011 and November 2013. The administrative regions in both the RCT and implementation studies contained both urban and rural areas. Turku was the only region that participated in both studies.

There were both differences and similarities between the implementation and the RCT intervention studies. First, the implementation group received the intervention when it was integrated as a normal practice of the child health clinics, and therefore, all families who met the inclusion criteria were eligible to enter. In the implementation phase, both participants and the health care workers received information that the SFSW parent training intervention has been evaluated as an intervention with strong documented effects by the Finnish national evaluation and classification system for evidence-based interventions [23]. This evaluation was partly based on the results of our previous RCT study [13,14]. In contrast, in the RCT, the intervention was not integrated as a normal practice of the child health clinics. Only those families who were randomized to the intervention group received the intervention. Second, in the implementation phase, an implementation plan, including decision supporting and administration component, was followed. This was important because the implementation phase included increasing number of communities in the whole Finland while the RCT was conducted in a predetermined area of Southwest Finland. Third, the most important similarity was that the content of the SFSW intervention was maintained in the implementation group as identical as possible with that of the original RCT intervention. In both groups, the same psychopathology and parenting measures were collected at baseline and 6-month follow-up. Data on children’s daily activities were collected only for the implementation group immediately after the intervention and at the 6-month follow-up. The timeline of the RCT and implementation studies is shown in Figure S1 of Multimedia Appendix 1. The study protocol of the RCT has previously been published [24] and registered at ClinicalTrials.gov (NCT01750996).

### Participants

This study focused on the 6-month follow-up assessments of children who displayed a high level of disruptive behavior when they were screened at 4 years of age during routine child health clinic visits. The screening procedure in the implementation study followed the same principles that were used in the RCT study. It was integrated into the standard 4-year-old child health checkups carried out by the child health clinics in the participating administrative regions [13]. All children living in Finland are invited to annual health assessments before they start school at 7 years of age, and attendance rates are just under 100% [25].

**Figure 1:**
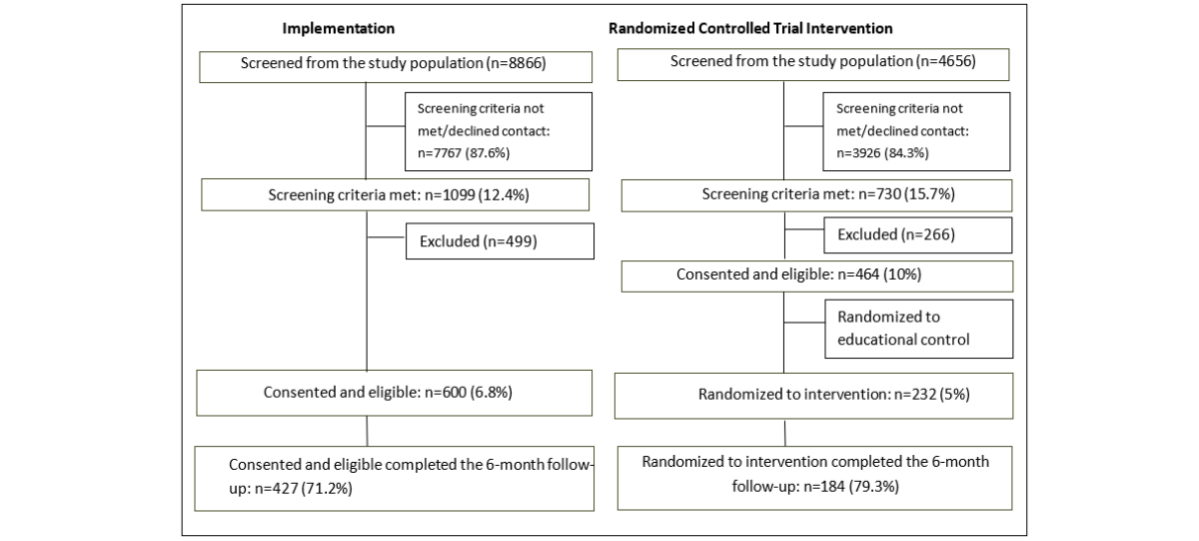
Flowchart of the families in the implementation and randomized controlled trial intervention groups.

In the implementation group, the first 600 eligible parents who agreed to take part in the program received the SFSW parent training intervention. Initially, 8866 children were screened for highly disruptive behavior and 1099 (12.4%) met the screening criteria. The implementation group equated to 6.8% (600/8866) of the initial population-based sample and 54.6% (600/1099) of those who were eligible to take part. The reference group consisted of 232 families who were randomized to receive the intervention during the previous RCT study [13,14]. Information was obtained from 427 (71.2%) of the 600 families in the implementation group at the 6-month follow-up assessments compared to 184 (79.3%) of the 232 families in the RCT intervention group. Figure 1 shows the flowchart of the implementation and RCT intervention groups. The families were typically recruited within 1 month of the child’s fourth birthday. They received a study information pack and were asked to bring the completed health questionnaire to the clinic.

### Inclusion and Exclusion Criteria

The screening measures and enrollment criteria were identical for the implementation and RCT studies [13]. Population-based screening for behavior problems was conducted for all children at the age of 4 years by using the conduct scale of the Strengths and Difficulties Questionnaire [26-28]. The parents were asked if their child had mild, moderate, or severe problems through a single question: “Overall, do you think that your child has difficulties in one or more of the following areas: emotions, behavior, or being able to get on with other people?” About 16.5% of the children (16.7% [1477/8866] and 16.3% [758/4656] in the implementation and RCT groups, respectively) who were screened, scored 5 or more out of 10 corresponding to the 80th percentile cutoff point and reported that the child had difficulties. This indicated a high level of behavior problems. The other inclusion criteria were that the parents perceived that child had at least minor difficulties in emotions, behavior, or social interactions. To participate in the study, the family had to live in an administrative region participating in the study, at least one parent had to speak native Finnish or Swedish, and they needed access to a telephone, computer, and internet connection. We excluded children who were unable to speak in full sentences, had hearing or vision impairments, or were receiving or had received behavior treatment. The exclusion criteria also included children who had been diagnosed with autism, Down syndrome, fetal alcohol syndrome, an intellectual disability, a severe mental disorder such as psychosis or depression, or who had a genetic diagnosis of mental retardation. We also excluded parents whose children did not live with them because they were subject to child protection services owing to child custody, abuse, or neglect issues. Details of the inclusion and exclusion criteria have previously been reported [24].

### Procedure

The participants in the implementation and RCT intervention study groups received the SFSW parent training program, which combines an interactive website with weekly telephone coaching [15,29]. One parent was identified for each child and they filled in the web-based questionnaire. However, they were also encouraged to get the child’s other parent involved in the program as much as possible. The program was guided by coaches who were professionals of health care and social services, that is, public health nurses, public nurses, or social workers from the child services. Of note, in the RCT study, there were 6 coaches, and during the implementation study, there were 10 coaches, 6 of whom had not participated in the RCT. The coaches had weekly phone calls with the parents, which were sometimes organized using texts or emails, and they monitored their progress on the website. The intervention consisted of 11 weekly themes that were explored during the interactive web-based program and the associated telephone coaching sessions. After the baseline survey, the coaches called the parents and they agreed to personalize goals tailored to individual behavior problems demonstrated by the child. The program aimed to reduce the problems identified by the parents by teaching them positive and practical parenting skills. During the first 7 weeks, the parent learned positive and practical problem-solving skills and were encouraged to develop an understanding of their child’s emotional development. The primary aim was to reorient the parent so that they noticed the child’s positive, not negative, behavior and reacted with a positive response. The second aim was to apply the skills in everyday situations, to plan daily activities in advance, and to use the methods they were taught to reinforce positive behavior. The final weekly themes focused on reinforcing their new skills and developing sustained positive parenting. The parents practiced the acquired skills with their child, independent of the coach’s support, and learned how to sustain the skills once the program had finished. The content and the conceptual framework of the weekly themes are depicted in Table 1. Each internet-based session comprised an introduction to the weekly theme, session content, video exercises, troubleshooting tips, and a review of what the parent had learnt. Instructional videos and audio clips illustrated the practical applications of the parents’ new skills. The coaches gave the parents feedback about their progress in applying the new skills and encouraged them throughout the program. They only proceeded to the next weekly theme when the parents had mastered the skill-related questions in the current one. This typically took 1 or 2 weeks. The children did not have access to the website or take part in the coaching calls. We are not aware of any potential adverse effects of the parent training in this study or in previous studies [30].

**Table 1:** The content and the conceptual framework of the skill training process of the Strongest Families Smart Website internet-based and telephone-assisted parent training intervention.

Session	Training components	Key training elements	Parental goals	Coaching elements	Parental action
Introduction to the program	Telephone coaching	Set up the parents for success	Reorient the parents to “How to break the negative circle”	Working alliance Identifying behavior problems Goal setting Present the first weekly theme	Actively start to notice the good
Notice the good	Web-based material (text, videos, audio clips) Telephone coaching	Positive and active parenting	Boost self-esteem of the child and parents and change the parents’ views of the child	Working alliance Evaluate the goal setting by modeling, practice such as role play, feedback, support	Notice good behavior often Positive verbal interaction and body language
Spread attention around	Web-based material (text, videos, audio clips) Telephone coaching	Positive, impartial parenting	Strengthen child’s empathy skills	Same as above	Learn to spread attention actively Praise the child for interacting positively with others
Ignore whining and complaining	Web-based material (text, videos, audio clips) Telephone coaching	Positive, self-controlled parenting	Teaches parents self-regulation	Same as above	Use positive thinking to stay calm and in control of the situations
Prepare for changes	Web-based material (text, videos, audio clips) Telephone coaching	Positive, proactive parenting	Reinforce good daily routines	Same as above	Warn that behavior must change Use positive “when you do this, then this will happen” statements
Plan ahead at home	Web-based material (text, videos, audio clips) Telephone coaching	Positive, proactive parenting	Reinforce child’s active role and involve them in planning	Same as above	Listens to the child’s ideas, plans daily situations at home
Reinforce by rewarding	Web-based material (text, videos) Telephone coaching	Positive, active parenting	Involve the child in planning and reinforce good daily routines	Same as above	Understand realistic goal setting and how to use praises and rewards
Plan ahead outside the home	Web-based material (text, videos) Telephone coaching	Positive, proactive parenting	Reinforce child’s active role and involve them in planning	Same as above	Listen to the child’s ideas Plan situations outside the home
Cooperate with day care	Web-based material (text, videos) Telephone coaching	Positive cooperation and communication between parent and day care	Help child to manage and succeed	Same as above	Set realistic goals and rewards Cooperate
Plan how to use time-out	Web-based material (text, videos) Telephone coaching	Positive, self-controlled parenting	Teach self-regulation and consistency	Reassure and use positive skills How to use time-out	Learn to be consequent Plan how to manage difficult situations
Revise: Problem-solving and future application of skills	Web-based material (text, videos) Telephone coaching	Positive daily parenting in future	Remind parents of positive proactive parenting skills	Ensure that parent is using all the skills and stays on track	Understand how using skills helps to prevent setbacks

### Quality Assurance and Implementation Plan

To ensure the integrity of the intervention and the accuracy of the data, several quality assurance measures were in effect during the implementation phase. These were similar to the quality assurance measures during the RCT study [13,14]. The implementation plan is summarized below and has been previously described in detail [15]. The implementation plan was driven by 3 core components [15,19]. First was recruitment, staff selection, and training. Once the coaches were recruited, they received intensive training on the SFSW program and were supervised and regularly monitored to make sure they adhered to the protocol. Together with supervision and staff performance evaluation, this provided systematic quality assurance [15]. The second core component was ongoing supervision and staff performance evaluation. The coaches took part in systematic weekly supervision meetings and group case conferences, where they reviewed and discussed the families they were coaching. Coaches with previous experience of the SFSW program acted as supervisors. After each telephone call, the coaches assessed their own performance on a scale of 4-10. The supervisor received a message from the digital platform about self-assessments that scored 6 or more and discussed the content of the call with the coach. To ensure the fidelity of the data, about 10% of the phone calls was audited by the coach supervisors with the parent’s permission and evaluated for competency. Additional training and monitoring of future calls were provided, if indicated. The coaches were required to report any adverse effects such as safety issues, abuse, or neglect to the supervisors, and the case was reported to the child protective services. Of note, 3 cases were reported during the implementation study and none during the RCT. The third core component was the decision supporting and administration. The development, delivery, and implementation process of the digital SFSW parent training intervention were centralized at the Research Center for Child Psychiatry at the University of Turku. The research group and the assisting staff of the Research Center introduced the SFSW intervention and the implementation process to the directors of child and family health services of the primary health care of each administrative region. A jointly funded research contract was signed by both parties. The research group maintained contact with the directors across the study region by organizing regular meetings and providing them with user-friendly monthly progress reports, which included the number of families who had been screened and enrolled. Training was offered to the team leaders of the child health clinics and public health nurses in order to integrate the intervention into primary health care. Moreover, local and national media were involved to increase public awareness of the SFSW intervention.

### Measures

#### Child Measures

The outcome measures were the same in the implementation and RCT studies [13,14]. The main measurement tool used to measure disruptive behavior was the 24-item Child Behavior Checklist 1.5-5 (CBCL/1.5-5) version for preschool children [31]. The CBCL/1.5-5 asks parents to rate emotional, behavioral, and social problems and has an additional section where they can provide extra information. It yields total scores and syndrome scales for the following items: emotionally reactive, anxious/depressed, somatic complaints, withdrawn, sleep problems, attention problems, and aggressive behavior. The first 4 syndromes yield the internalizing score, while the last 2 yield the externalizing score. The CBCL/1.5-5 also includes 5 subscores from the Diagnostic and Statistical Manual of Mental Disorders, fifth edition: affective, anxiety, pervasive developmental problems, attention-deficit/hyperactivity disorder, and oppositional disorder [32]. A large cross-cultural study from 24 countries, including Finland, reported good psychometric properties and good internal consistency for the CBCL preschool version (Cronbach alphas for total, externalizing, and internalizing scores: .94, .88, and .84, respectively) [33,34]. We used the Inventory of Callous-Unemotional Traits (ICU) to measure child psychopathy traits. The instrument consists of 24 items and has been reported to have good psychometric properties for 4-year-old children [35,36]. Cronbach alphas of .93, .81, .88, and .86 have previously been reported for total score, callousness, uncaring, and unemotional scores, respectively, for 4-year-old children [35].

Daily activities were only assessed for the implementation study. Parents were asked to rate the impact of the child´s behavior during daily transitions, including getting dressed, getting ready for day care, during the evening meal, and getting ready for bed. It also covered social interactions, including playing with siblings and other children during a car or bicycle ride and in public places such as the supermarket. A Cronbach alpha of .64 was calculated using our implementation data. The questionnaire was adapted from the Barkley Home Situations Questionnaire, which asks the parent to rate whether the child’s behavior causes problems during specified daily routines [37].

#### Parent Measures

The Parenting Scale, which is a 30-item questionnaire, was used to measure parenting skills [38,39]. Cronbach alphas of .78, .66, .68, and .50 were calculated for total score, laxness, overreactivity, and hostility, respectively, by using our implementation data. We evaluated the parents’ stress, anxiety, and depression symptoms with the shorter 21-item Depression, Anxiety and Stress Scale (DASS-21) [40]. The internal consistency of DASS-21 has been reported as 0.93, 0.88, 0.82, and 0.90 for total scale and DASS-21, respectively, in a large study that represented a nonclinical sample [41].

### Statistical Analyses

The analyses compared the 600 families in the real-world implementation group to the 232 families in the RCT intervention group. Categorical demographic variables, including the child, parent, and family characteristics, are presented as numbers and percentages. Continuous demographic variables, including the parents’ age and duration of child’s behavioral problems, are presented as means and standard deviations. We explored any differences at baseline between the 2 groups by using Pearson chi-square test or Fisher exact test for the categorical variables and the two-tailed Student *t* test for the continuous variables. The primary and secondary outcome variables were analyzed with a linear mixed-effect model for repeated measurements. The within factor was time, namely, baseline and 6-months follow-up, and the RCT intervention group and the implementation group provided the between factor. The covariates in the statistical models were CBCL externalizing scores at baseline, maternal education, duration of behavior problems, and the baseline measurement of each outcome. The statistical model used to analyze the CBCL externalizing score consisted of the group and time main effects, the group-by-time interaction effect, and the following covariates: the CBCL externalizing score at baseline, maternal education, and duration of behavior problems. Meanwhile, the statistical model used to analyze all the secondary outcome variables, namely, the CBCL total and other CBCL subscores, ICU, the Parenting Scale, and DASS-21 consisted of the group and time main effects and the group-by-time interaction effect. It also included the following covariates: the specific secondary variable to be analyzed at baseline, the CBCL externalizing score at baseline, maternal education, and the duration of behavior problems.

The sensitivity analyses comprised the families who had completed the parent training program as well as the treatment comparisons. Turku was excluded from analysis, as it was the only site that had taken part in both the implementation and RCT intervention studies. As the study subjects in the implementation group were recruited from January 2015 to May 2017 and in the RCT intervention group from October 2011 to November 2013, we also tested the effect of the recruitment year on the CBCL externalizing score at baseline. The model included the effects of recruitment year, maternal education, and duration of behavior problems. The effect of the recruitment year was insignificant (*P*=.17). An additional analysis also compared the implementation to the RCT education control group. The model included the CBCL externalizing score at baseline, maternal education, duration of behavior problems, paternal age, and the baseline measurements of each outcome as covariates. A *P* value <.05 was considered to be statistically significant. The statistical analyses were performed using SAS 9.4 (SAS Institute).

### Ethics Approval

Ethical approval for the implementation study was received from the University of Turku (approval number: 18/2018). The parents provided written informed consent for both the implementation and the RCT studies.

## Results

**Figure 2:**
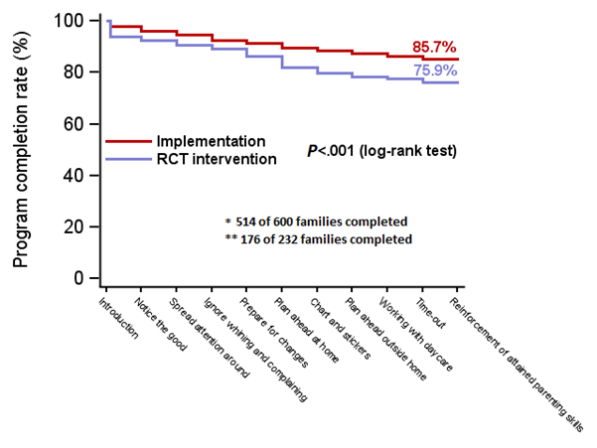
Kaplan-Meier curves of families completing the program in the implementation and the randomized controlled trial intervention groups. RCT: randomized controlled trial, fixed axes according to editor comments.

The number of families who discontinued the program was 86 (14.3%) of the 600 families in the implementation group compared to 56 (24.1%) of the 232 families in the RCT intervention group. This meant that the odds ratio was 1.9 with a 95% CI of 1.3 to 2.8 (*P*<.001), as seen in Figure 2. The 6-month follow-up assessment was completed by 71.2% (427/600) of the parents in the implementation group and 79.3% (184/232) of the parents in the RCT intervention group (*P*<.001), as seen in Figure 1. Table 2 shows that there were no differences between the implementation group and the RCT intervention group when it came to the parent, family, and child characteristics and the factors related to parent training program. However, the mothers in the implementation group had higher educational levels than the mothers in the RCT intervention group (*P*=.046) and the children experienced a longer duration of behavior problems (*P*=.004). The mean duration of the telephone coaching calls was 37 minutes in both the implementation and the RCT intervention groups. The total duration of telephone coaching plus the average time spent on the program website was 13.8 hours in the implementation group and 14.1 hours in the RCT intervention group (*P*=.49).

**Table 2:** Demographic characteristics of the families and treatment factors in the implementation and the randomized controlled trial intervention groups.

Demographics				Implementation group (n=600)		Randomized controlled trial intervention (n=232)		*P* value
**Parent and family characteristics**								
	**Family structure^a^, n (%)**							.54
		Two biological parents	489 (81.6)		191 (83.5)			
		Single biological parent	82 (13.7)		24 (10.4)			
		Biological parent and foster parent	19 (3.2)		9 (3.9)			
		Other	9 (1.5)		5 (2.2)			
	**Age (years), mean (SD)**							
		Maternal	30.3 (4.8)		30.5 (5.4)		.68	
		Paternal	32.7 (5.7)		33.2 (5.9)		.28	
	**Maternal education^b^, n (%)**							.046
		Elementary school or less	15 (2.5)		13 (5.7)			
		Secondary education	204 (34.2)		85 (37)			
		College or university degree	378 (63.3)		132 (57.4)			
	**Paternal education^c^, n (%)**							.28
		Elementary school or less	27 (4.8)		16 (7.4)			
		Secondary education	280 (50.1)		99 (45.8)			
		College or university degree	252 (45.1)		101 (46.8)			
**Child characteristics, n (%)**								
	**Sex**							.82
		Female	238 (39.7)		90 (38.8)			
		Male	362 (60.3)		142 (61.2)			
	**Day care outside home^d^**							.29
		Yes	476 (79.9)		192 (83.1)			
		No	120 (20.1)		39 (16.9)			
	**Behavioral problems**							.18
		Minor	301 (50.2)		129 (55.6)			
		Definite	252 (42)		92 (39.7)			
		Severe	47 (7.8)		11 (4.7)			
	**Duration of problems^e^**							.004
		<6 months	193 (33)		102 (45.1)			
		6-12 months	155 (26.5)		44 (19.5)			
		>12 months	237 (40.5)		80 (35.4)			
**Program characteristics, mean (SD)**								
	Total number of calls			10.4 (2.5)		10.1 (3.3)		.20
	Duration of calls for the 11 themes (min)			37.3 (11.0)		37.3 (13.5)		.96
	Duration of website access per theme (min)			45.3 (19.3)		47.8 (19.9)		.12
	Total duration of calls (h)			6.5 (2.4)		6.4 (3.3)		.65
	Total duration of website access (h)			7.3 (2.8)		7.5 (3.2)		.56
	Total duration of program (h)			13.8 (4.3)		14.1 (5.4)		.49

^a^Missing observations: implementation group (n=1); randomized controlled trial group (n=2).

^b^Missing observations: implementation group (n=3); randomized controlled trial group (n=2). Pairwise comparisons: elementary school or less versus secondary education (*P*=.06); elementary school or less versus college or university degree (*P*=.02); secondary education versus college or university degree (*P*=.28).

^c^Missing observations: implementation group (n=41); randomized controlled trial group (n=1).

^d^Missing observations: implementation group (n=4); randomized controlled trial group (n=1).

^e^Missing observations: implementation group (n=15); randomized controlled trial group (n=6). Pairwise comparisons: <6 months versus 6-12 months (*P*=.003); <6 months versus >12 months (*P*=.01); 6-12 months versus >12 months (*P*=.42).

In the implementation group, there were significant improvements from the baseline to the 6-month follow-up assessment in the primary outcome, which was the CBCL externalizing score. The same was true for the secondary outcomes: CBCL total and internalizing scores and the total scores of the ICU, Parenting Scale, and DASS-21 (Table 3). The sensitivity analysis, which included the participants who completed the whole program (Table S1 of Multimedia Appendix 2), yielded similar estimates of the improvements in all the outcomes. Table 4 shows the mean scores of the primary outcome, CBCL externalizing score, and the secondary outcomes at baseline and 6 months in the implementation and the RCT intervention groups. There were no significant differences between the 2 groups in the CBCL externalizing, total, or internalizing scores. In addition, no significant differences were seen in the total scores of the Parenting Scale or ICU. The estimated difference of 2.5 (95% CI 0.0-5.1) points in DASS-21 nearly reached statistical significance (*P*=.05), indicating better improvement in the implementation group when it was compared to that of the RCT intervention group. Of note, the improvement in DASS-21 showed significantly better improvement in the implementation group (estimated difference 1.1, 95% Cl 0.1-2.2; *P*=.04). When only the participants who completed the whole parent training program in the implementation group were compared to those in the RCT intervention group, the results remained similar (Table S2 of Multimedia Appendix 3).

The additional analyses compared the changes in primary and secondary outcomes between the implementation and the RCT education control groups, as shown in Table S3 of Multimedia Appendix 4. There were significant differences between the groups in CBCL externalizing, total, and internalizing scores, as well as the total scores of the Parenting Scale and DASS-21. However, the total ICU score did not reach statistical significance (*P*=.27). As the city of Turku participated in both the implementation study and the RCT study, we repeated the analyses by excluding the participants living in Turku from the implementation group. This did not show any significant differences in any of the symptom scores between the study groups (Table S4 of Multimedia Appendix 5). Changes in daily activities from the baseline assessment to posttreatment and the 6-month follow-up assessment are shown in the Table S5 of Multimedia Appendix 6. This information was only obtained from the implementation group; therefore, comparisons with the RCT intervention group could not be made. There were significant improvements in all measurements for social interactions and daily transitions from baseline to posttreatment and to the 6-month follow-up. The data for daily activities were obtained from 83% (498/600) of the participants in posttreatment and 66.5% (399/600) of the participants in the follow-up.

**Table 3:** Change from baseline to 6 months in child psychopathology, parenting skills, and parents’ stress in the implementation group.

Variable					Baseline (n=600), mean^a^ (SE)	After 6 months (n=600), mean^a^ (SE)		Mean change^b^ (SE)		95% CI		*P*^c^ value
**Child measures**												
	**Primary outcome**											
		Child Behavior Checklist externalizing score		21.1 (0.5)		14.8 (0.5)	6.2 (0.4)		5.5 to 7.0		<.001	
	**Secondary outcomes**											
		Child Behavior Checklist Total score		48.8 (1.2)		33.6 (1.3)	15.2 (1.0)		13.3 to 17.2		<.001	
		Child Behavior Checklist Internalizing score		12.1 (0.4)		8.5 (0.5)	3.6 (0.4)		2.9 to 4.3		<.001	
		**Symptom domains**										
			Aggression	18.0 (0.4)		12.5 (0.4)	5.5 (0.3)		4.9 to 6.1		<.001	
			Attention	3.1 (0.1)		2.4 (0.1)	0.7 (0.1)		0.6 to 1.0		<.001	
			Sleep	4.0 (0.2)		2.5 (0.2)	1.5 (0.1)		1.2 to 1.7		<.001	
			Withdrawn	2.4 (0.1)		1.6 (0.1)	0.8 (0.1)		0.6 to 1.0		<.001	
			Somatic	2.9 (0.1)		2.0 (0.2)	0.8 (0.1)		0.6 to 1.1		<.001	
			Anxious	2.9 (0.1)		2.0 (0.1)	0.8 (0.1)		0.6 to 1.0		<.001	
			Emotional	3.9 (0.2)		2.8 (0.2)	1.2 (0.1)		0.9 to 1.4		<.001	
		**Diagnostic and Statistical Manual of Mental Disorders, fifth edition subscores**										
			Affective problems	3.3 (0.1)		2.0 (0.2)	1.3 (0.1)		1.1 to 1.5		<.001	
			Anxiety problems	4.2 (0.2)		2.9 (0.2)	1.4 (0.1)		1.1 to 1.6		<.001	
			PDD^d^ problems	4.7 (0.2)		3.3 (0.2)	1.4 (0.2)		1.1 to 1.7		<.001	
			ADHD^e^ problems	6.0 (0.2)		4.5 (0.2)	1.6 (0.1)		1.3 to 1.8		<.001	
			ODD^f^ problems	6.5 (0.2)		4.6 (0.2)	1.9 (0.1)		1.6 to 2.1		<.001	
		**Inventory of Callous-Unemotional Traits**										
			Total	24.6 (0.5)		20.6 (0.5)	4.0 (0.4)		3.2 to 4.7		<.001	
			Callousness	8.3 (0.2)		6.2 (0.2)	2.2 (0.2)		1.8 to 2.5		<.001	
			Uncaring	13.2 (0.2)		11.6 (0.3)	1.6 (0.2)		1.3 to 2.0		<.001	
			Unemotional	3.1 (0.1)		2.9 (0.1)	0.2 (0.1)		–0.1 to 0.4		.30	
**Parent measures**												
	**Parenting scale**											
		Total		3.2 (0.0)		2.7 (0.0)	0.6 (0.0)		0.5 to 0.6		<.001	
		Laxness		2.7 (0.0)		2.2 (0.0)	0.4 (0.1)		0.4 to 0.5		<.001	
		Overreactivity		3.9 (0.1)		3.1 (0.1)	0.8 (0.0)		0.7 to 0.9		<.001	
		Hostility		1.9 (0.0)		1.6 (0.1)	0.3 (0.1)		0.3 to 0.4		<.001	
	**21-item Depression, Anxiety and Stress Scale short form**											
		Total		18.5 (1.1)		12.1 (1.1)	6.4 (0.8)		4.9 to 7.9		<.001	
		Depression		5.2 (0.5)		3.1 (0.5)	2.1 (0.3)		1.4 to 2.7		<.001	
		Anxiety		2.4 (0.3)		1.4 (0.3)	1.1 (0.2)		0.7 to 1.4		<.001	
		Stress		11.0 (0.5)		7.7 (0.5)	3.3 (0.4)		2.6 to 4.0		<.001	

^a^Least-squares means.

^b^Change from baseline to 6 months after providing informed consent.

^c^Adjusted with maternal education and duration of problems.

^d^PDD: pervasive developmental disorder.

^e^ADHD: attention-deficit/hyperactivity disorder.

^f^ODD: oppositional defiant disorder.

**Table 4:** Mean changes from baseline to 6 months in child psychopathology, parenting skills, and parents’ stress in the implementation and randomized controlled trial intervention groups.

Variable			Mean (SE) change from baseline to 6 months				Implementation versus RCT intervention, mean (95% CI)		*P*^c^ value	
			Implementation group (n=600), mean^a^ (SE)		RCT^b^ intervention (n=232), mean^a^ (SE)					
**Child measures**										
	**Primary outcome**									
		Child Behavior Checklist externalizing score	6.3 (0.4)		6.1 (0.6)		–0.2 (–1.3 to 1.6)		.83	
	**Secondary outcomes**									
		Child Behavior Checklist total score	15.3 (1.0)		14.6 (1.6)		–0.7 (–3.0 to 4.5)		.70	
		Child Behavior Checklist internalizing score	3.7 (0.4)		3.4 (0.6)		–0.3 (–1.0 to 1.6)		.64	
	**Symptom domains**									
		Aggression	5.5 (0.3)		5.5 (0.5)		–0.0 (–1.2 to 1.3)		.95	
		Attention	0.7 (0.1)		0.6 (0.1)		–0.1 (–0.2 to 0.4)		.53	
		Sleep	1.5 (0.1)		1.5 (0.2)		–0.0 (–0.5 to 0.5)		1.0	
		Withdrawn	0.8 (0.1)		0.5 (0.2)		–0.3 (–0.0 to 0.7)		.08	
		Somatic	0.8 (0.1)		0.6 (0.2)		–0.2 (–0.2 to 0.7)		.29	
		Anxious	0.9 (0.1)		1.0 (0.2)		–0.1 (0.5 to 0.3)		.62	
		Emotional	1.2 (0.1)		1.3 (0.2)		–0.1 (–0.7 to 0.4)		.58	
	**Diagnostic and Statistical Manual of Mental Disorders, fifth edition subscores**									
		Affective problems	1.3 (0.1)		1.3 (0.2)		0.0 (–0.4 to 0.5)		.95	
		Anxiety problems	1.4 (0.1)		1.5 (0.2)		–0.1 (–0.6 to 0.4)		.69	
		PDD^d^ problems	1.4 (0.2)		1.2 (0.3)		0.2 (–0.3 to 0.8)		.41	
		ADHD^e^ problems	1.6 (0.1)		1.2 (0.2)		3.5 (–0.2 to 0.9)		.17	
		ODD^f^ problems	1.9 (0.1)		2.2 (0.2)		–0.3 (–0.7– 0.2)		.26	
	**Inventory of Callous-Unemotional Traits**									
		Total	4.0 (0.4)		4.3 (0.7)		–0.4 (–1.9 to 1.2)		.64	
		Callousness	2.0 (0.2)		2.1 (0.3)		–0.1 (–0.7– 0.8)		.83	
		Uncaring	1.6 (0.2)		1.9 (0.3)		–0.2 (–1.0 to 0.5)		.53	
		Unemotional	0.2 (0.1)		0.3 (0.2)		–0.1 (–0.6 to 0.3)		.44	
**Parent measures**										
	**Parenting scale**									
		Total	0.6 (0.0)		0.5 (0.0)		0.0 (–0.1 to 0.1)		.50	
		Laxness	0.4 (0.0)		0.4 (0.1)		0.0 (–0.1 to 0.2)		.79	
		Overreactivity	0.8 (0.1)		0.6 (0.1)		0.2 (–0.0 to 0.4)		.07	
		Hostility	0.3 (0.0)		0.3 (0.1)		0.0 (–0.1 to 0.2)		.85	
	**21-item Depression, Anxiety and Stress Scale short form**									
		Total	6.4 (0.7)		3.9 (1.1)		2.5 (0.0 to 5.1)		.05	
		Depression	2.1 (0.3)		1.0 (0.5)		1.1 (0.1 to 2.2)		.036	
		Anxiety	1.0 (0.2)		0.8 (0.3)		0.3 (–0.4 to 0.1)		.44	
		Stress	3.3. (0.4)		2.2 (0.6)		1.1 (–0.2 to 2.4)		.09	

^a^Least-squares means.

^b^RCT: randomized controlled trial.

^c^Adjusted with maternal education and duration of problems.

^d^PDD: pervasive developmental disorder.

^e^ADHD: attention-deficit/hyperactivity disorder.

^f^ODD: oppositional defiant disorder.

## Discussion

This was the first population-based study to evaluate the effectiveness of an internet-based and telephone-assisted parent training intervention for children with behavior problems when it was implemented in real-world practice. The children’s psychiatric problems improved, including externalizing and internalizing problems and callousness. The findings were remarkable from the perspective of the children’s social development, as the program had significant effects on daily transitions and activities such as getting dressed, dining behavior, activities outside the home, and interactions with other people. Parents reported that their parenting skills had improved and they demonstrated less distress in dealing with their children at the 6-month follow-up. Most importantly, this study shows that the improvements that had been achieved were similar to those reported for the intervention group in the RCT. There was no difference in the changes in the children’s psychiatric problems or parenting skills when the implementation and RCT groups were compared. Furthermore, when changes between the implementation and RCT education control groups were compared, the implementation group showed significantly better improvements in the children’s externalizing and internalizing problems as well as in parenting skills and parents’ distress. In addition to the effectiveness of the treatment, the ability to engage and retain parents in the program is one of the keys to successful parent training interventions [42-44]. Previously, we reported high parental satisfaction levels in both the RCT and implementation groups [15]. High satisfaction levels and the quality of relationships between parents and professionals have been associated with greater improvements in the effectiveness of interventions [45,46]. The dropout rate in our RCT study was 24%, while previous studies on digital parenting interventions report usually 30%-50% dropout rates [12,47-50]. In general, high dropout rates in digital interventions have been especially associated with nonguided interventions [43,51-54]. The reasons for the exceptionally low dropout rate in the implementation phase (14%) are likely to be multiple. One possible explanation is that in the implementation phase, the SFSW intervention that was offered had gained research-based evidence and the benefits of it were known and communicated to the professionals in the primary health care, especially in child health clinics, and to parents and largely in the media. Thus, the public and the professionals were aware of the intervention and its benefits. It is very likely that this convinced both health nurses at the child health clinics who motivate the parents in engaging in the program and the concerned parents tackling with their child’s challenges.

In order to successfully implement interventions, we need to know whether they work and *why* they work [19]. Success can be related to how appropriate the background theory is, the context where the intervention takes place, practical issues such as how easy it is to attend sessions, and specific intervention practices such as practicing specific parenting skills [55]. Our SFSW intervention fulfilled these criteria well. It was based on the social learning theory and the cognitive behavioral theory as well as principles of positive parenting, which provided a sound theoretical framework for the intervention. The context of the program was well-defined, including a clear definition of the population that the program was aimed at, and there were clear inclusion and exclusion criteria. The program also had a clear structure, including a description of the core components, which was practiced through modeling, practice, feedback, and support. It has previously been emphasized that a solid framework and a structured implementation plan are needed to successfully make the transition from evidence-based psychosocial interventions to *real-world* clinical practice [22]. We systematically followed a structured plan during the implementation process [15]. The SFSW program contained the core implementation drivers that facilitated the process when intervention was implemented in the primary health care. The same quality assurance measures were in place during the RCT and implementation phase. These were based on the centralized delivery of the intervention, which used a digital platform and ongoing training, monitoring, and supervision of the program coaches. It is important to note that the primary health care staff were also provided with ongoing training. In addition, the program was effectively administered by including regular meetings with the directors of the child and family services and providing them with user-friendly reports. Media coverage raised awareness, and this made it easier to recruit families and increased the perceived value of the program [15].

Several practical features of the program may have paved the way for positive outcomes during the real-world implementation. First, the program was much easier for the parents than face-to-face interventions because they did not need to leave home or work or make childcare arrangements. Second, the telephone coaching provided immediate problem-solving, which may have been more rewarding for the parents than communicating using emails or text messages. A recent meta-analysis showed that digital interventions that included support and guidance, such as telephone calls, had larger effect sizes on mental health outcomes than smartphone interventions without any personal support [56]. Third, the coaches were well-trained and formed good relationships with the parents [15], which is central to the success of any intervention [57].

There were some limitations in our study. First, although the parental and child outcomes were measured using well-validated questionnaires, they were rated by the same person, namely, the parent. One parent was identified for each child, but they were also encouraged to get the child’s other parent involved in the program as much as possible. Further details on the level of parental involvement could have added to the richness of the data, but there were practical limitations to collecting this. To reduce the possibility of the common rater variance, observations by other informants such as day care personnel could have validated our findings. Second, we have discussed mechanisms that could have been responsible for the positive outcomes. However, there is very little empirical evidence on whether the effects of the intervention resulted from the internet sessions, the personal telephone coaching, parental motivation, or a combination of those factors. Further studies need to examine factors that explain these positive outcomes. Personalized medicine is increasingly being used to move away from one-size-fits-all interventions to those that are more tailored to individual needs. This approach could yield useful information on the mechanisms underlying interventions and enable more accurate targeting.

The target group, content, and effectiveness of the intervention were maintained when the implementation group results were compared with the findings of the RCT intervention. Internet-based telephone-assisted parent training interventions may have advantages over traditional group-based treatment approaches when the goal is to identify children at risk in the community at an early stage. This new approach can provide effective parent training for a large number of families, including many who would not normally participate in clinic-based services. Referring families who need parent training to clinical services often results in substantial delays and they need other support while they are waiting. Digitally delivered interventions move child mental health treatment outside traditional clinics and into people’s homes and schools, increasing access and reducing stigma. In addition, they can be increased to help more families, and parents are more likely to stay with the program until the end. There is a global shortage of skilled staff who can address child mental health problems in low- and high-income countries and even in countries with public health care [58,59]. This could become an even greater issue when demand inevitably increases because of the impact of the COVID-19 pandemic on children and the effects of the expected global recession on health care budgets. Our study highlights the positive findings that were demonstrated when our internet-based training and phone coaching initiative provided support for the parents of children with behavior problems, who were identified using population-based screening at primary health care. This initiative made the successful transition from an RCT to real-world settings, and our findings may have potential global implications for addressing the unmet needs of children with mental health issues if the findings are repeated in other sociocultural contexts.

## Appendix

Multimedia Appendix 1 Timeline of the randomized controlled trial and implementation studies.

Multimedia Appendix 2 Change from baseline to 6 months in child psychopathology, parenting skills and parents’ stress in the Implementation group for participants who completed the program.

Multimedia Appendix 3 Mean changes from baseline to 6 months in child psychopathology, parenting skills, and parents’ stress in the implementation and the randomized controlled trial intervention groups for participants who completed the program.

Multimedia Appendix 4 Mean changes from baseline to 6 months in child psychopathology, parenting skills, and parents’ stress in the implementation and randomized controlled trial educational control groups.

Multimedia Appendix 5 Change from baseline to 6 months in child psychopathology, parenting skills, and parents’ stress in the implementation and randomized controlled trial intervention groups. The city of Turku is excluded from the implementation data.

Multimedia Appendix 6 Change from baseline to posttreatment and 6 months in daily activities and social interactions in the implementation group (n=600).

## Abbreviations

CBCL: Child Behavior Checklist
DASS-21: 21-item Depression, Anxiety and Stress Scale
ICU: Inventory of Callous-Unemotional Traits
RCT: randomized controlled trial
SFSW: Strongest Families Smart Website
