# Supplementary material for: Effectiveness of an Internet-Based and Telephone-Assisted Training for Parents of 4-Year-Old Children With Disruptive Behavior: Implementation Research
Source: J Med Internet Res. 2022 Apr 4;24(4):e27900. doi: 10.2196/27900 (PMC9016503; doi:10.2196/27900)
Supplement: Multimedia Appendix 1 [file jmir_v24i4e27900_app1.docx]

**Figure S1. Timeline of the RCT and the implementation studies.**

| **2009** | **2010** | **2011** | **2012** | **2013** | **2014** | **2015** | **2016** | **2017** | **2018** | **2019** |
| --- | --- | --- | --- | --- | --- | --- | --- | --- | --- | --- |
| Program development and pilot study | | Randomized controlled trial: recruitment, intervention and follow up | | | Implementation plan, recruitment, intervention and follow up for the study. | | | | | |
