# Supplementary material for: Effectiveness of an Internet-Based and Telephone-Assisted Training for Parents of 4-Year-Old Children With Disruptive Behavior: Implementation Research
Source: J Med Internet Res. 2022 Apr 4;24(4):e27900. doi: 10.2196/27900 (PMC9016503; doi:10.2196/27900)
Supplement: Multimedia Appendix 2 [file jmir_v24i4e27900_app2.docx]

**Table S1.** Change from baseline to 6 months in child psychopathology, parenting skills and parents’ stress in the Implementation group for participants who completed the program.

| Variable | Baseline Mean^a^ (SE)  (n=514) | 6 months  Mean^a^ (SE)  (n=514) | Mean change^b^ (SE) | 95% CI | *P* value^c^ |
| --- | --- | --- | --- | --- | --- |
| ***Child measures*** | | | | | |
| **Primary outcome** | | | | | |
| CBCL^d^ Externalizing | 21.1 (0.6) | 15.2 (0.6) | 5.9 (0.4) | 5.1 to 6.7 | <.001 |
| **Secondary outcomes** | | | | | |
| CBCL^d^ Total | 47.6 (1.6) | 33.6 (1.6) | 14.0 (1.1) | 11.9 to 16.0 | <.001 |
| CBCL^d^ Internalizing | 11.4 (0.6) | 8.0 (0.6) | 3.4 (0.4) | 2.6 to 4.1 | <.001 |
| Symptom domains | | | | | |
| Aggression | 18.1 (0.5) | 12.7 (0.6) | 5.3 (0.3) | 4.6 to 6.0 | <.001 |
| Attention | 3.1 (0.1) | 2.5 (0.1) | 0.6 (0.1) | 0.4 to 0.8 | <.001 |
| Sleep | 3.8 (0.2) | 2.5 (0.2) | 1.3 (0.1) | 1.1 to 1.6 | <.001 |
| Withdrawn | 2.3 (0.2) | 1.6 (0.2) | 0.8 (0.1) | 0.6 to 1.0 | <.001 |
| Somatic | 2.6 (0.2) | 1.8 (0.2) | 0.7 (0.1) | 0.5 to 1.0 | <.001 |
| Anxious | 2.6 (0.2) | 1.9 (0.2) | 0.8 (0.1) | 0.5 to 1.0 | <.001 |
| Emotional | 3.8 (0.2) | 2.7 (0.2) | 1.1 (0.1) | 0.8 to 1.4 | <.001 |
| DSM*-*5 subscores | | | | | |
| Affective problems | 3.2 (0.2) | 2.0 (0.2) | 1.2 (0.1) | 0.9 to 1.4 | <.001 |
| Anxiety problems | 4.0 (0.2) | 2.7 (0.2) | 1.3 (0.1) | 1.0 to 1.6 | <.001 |
| PDD problems | 4.6 (0.2) | 3.2 (0.3) | 1.3 (0.2) | 1.0 to 1.6 | <.001 |
| ADHD problems | 6.0 (0.2) | 4.6 (0.2) | 1.3 (0.4) | 1.1 to 1.6 | <.001 |
| ODD problems | 6.6 (0.2) | 4.8 (0.2) | 1.8 (0.1) | 1.6 to 2.1 | <.001 |
| ICU^f^ | | | | | |
| Total | 24.6 (0.6) | 20.9 (0.6) | 3.7 (0.4) | 2.9 to 4.5 | <.001 |
| Callousness | 8.4 (0.3) | 6.4 (0.3) | 2.1 (0.2) | 1.7 to 4.5 | <.001 |
| Uncaring | 13.2 (0.3) | 11.7 (0.3) | 1.5 (0.2) | 1.1 to 1.9 | <.001 |
| Unemotional | 3.0 (0.2) | 2.9 (0.2) | 0.1 (0.1) | –0.1 to 0.4 | .30 |
| ***Parent measures*** | | | | | |
| Parenting scale^e^ | | | | | |
| Total | 3.3 (0.0) | 2.7 (0.0) | 0.6 (0.0) | 0.5 to 0.6 | <.001 |
| Laxness | 2.7 (0.1) | 2.3 (0.1) | 0.4 (0.0) | 0.3 to 0.5 | <.001 |
| Overreactivity | 3.9 (0.1) | 3.0 (0.1) | 0.8 (0.0) | 0.7 to 0.9 | <.001 |
| Hostility | 1.8 (0.1) | 1.5 (0.1) | 0.3 (0.0) | 0.3 to 0.4 | <.001 |
| DASS-21^g^ | | | | | |
| Total | 17.9 (1.3) | 11.8 (1.3) | 6.1 (0.8) | 4.5 to 7.6 | <.001 |
| Depression | 5.0 (0.6) | 3.1 (0.6) | 1.9 (0.3) | 1.3 to 2.6 | <.001 |
| Anxiety | 2.4 (0.4) | 1.4 (0.4) | 1.0 (0.2) | 0.6 to 1.4 | <.001 |
| Stress | 10.6 (0.7) | 7.4 (0.7) | 3.2 (0.4) | 2.4 to 4.0 | <.001 |

*Note*: ADHD = attention-deficit/hyperactivity disorder; ODD = oppositional defiant disorder; PDD = pervasive developmental disorder

^a^Least-squares means^; b^Change from baseline to 6 months after providing informed consent; ^c^Adjusted with maternal education and duration of problems; ^d^CBCL = Child Behavior Checklist; ^e^Missing observations n=8; ^f^ICU= Inventory of Callous-Unemotional Traits. Missing observations n=3; ^g^DASS-21= Depression Anxiety and Stress Scale Short Form, SE = standard error.
