# Supplementary material for: Effectiveness of an Internet-Based and Telephone-Assisted Training for Parents of 4-Year-Old Children With Disruptive Behavior: Implementation Research
Source: J Med Internet Res. 2022 Apr 4;24(4):e27900. doi: 10.2196/27900 (PMC9016503; doi:10.2196/27900)
Supplement: Multimedia Appendix 3 [file jmir_v24i4e27900_app3.docx]

**Table S2.** Mean changes from baseline to 6 months in child psychopathology, parenting skills and parents’ stress in the Implementation and the Randomized controlled trial (RCT) intervention groups for participants who completed the program.

| Variable | Mean (SE) Change From Baseline to 6 Months | | Implementation versus RCT intervention  mean (95% CI) | *P* ^b^ value |
| --- | --- | --- | --- | --- |
|  | Implementation mean^a^ (SE)  (n=514) | RCT intervention  mean^a^ (SE)  (n=176) |  |  |
| ***Child measures*** | | | | |
| **Primary outcome** | | | | |
| CBCL^c^ Externalizing | 5.9 (0.4) | 5.4 (0.7) | 0.5 (–1.1 to 2.1) | .54 |
| **Secondary outcomes** | | | | |
| CBCL^c^ Total | 14.0 (1.1) | 12.7 (1.8) | 1.3 (–2.9 to 5.5) | .55 |
| CBCL^c^ Internalizing | 3.4 (0.4) | 2.8 (0.7) | 0.6 (–0.9 to 2.1) | .43 |
| Symptom domains | | | | |
| Aggression | 5.3 (0.4) | 4.9 (0.6) | 0.4 (–1.0 to 1.8) | .57 |
| Attention | 0.6 (0.1) | 0.5 (0.2) | 0.1 (–0.3 to 0.5) | .55 |
| Sleep | 1.3 (0.1) | 1.4 (0.2) | 0.0 (–0.6 to 0.5) | .92 |
| Withdrawn | 0.8 (0.1) | 0.4 (0.2) | 0.3 (–0.1 to 0.7) | .12 |
| Somatic | 0.7 (0.1) | 0.5 (0.2) | 0.3 (–0.2 to 0.8) | .26 |
| Anxious | 0.8 (0.1) | 0.8 (0.2) | –0.1 (–0.5 to 0.4) | .89 |
| Emotional | 1.1 (0.1) | 1.0 (0.2) | –0.1 (–0.5 to 0.6) | .85 |
| DSM-5 subscores | | | | |
| Affective problems | 1.2 (0.1) | 1.2 (0.2) | –0.0 (–0.5 to 0.5) | .87 |
| Anxiety problems | 1.3 (0.2) | 1.4 (0.3) | –0.2 (–0.7 to 0.4) | .59 |
| PDD problems | 1.3 (0.2) | 1.1 (0.3) | 0.2 (–0.4 to 0.9) | .49 |
| ADHD problems | 1.4 (0.2) | 1.0 (0.3) | 0.4 (–0.2 to 0.9) | .22 |
| ODD problems | 1.8 (0.1) | 2.0 (0.2) | –0.2 (–0.7 to 0.4) | .55 |
| ICU^d^ | | | | |
| Total | 3.7 (0.4) | 3.8 (0.7) | –1.1 (–1.8 to 1.6) | .90 |
| Callousness | 2.1 (0.2) | 1.7 (0.4) | 0.4 (–0.4 to 1.2) | .32 |
| Uncaring | 1.5 (0.2) | 1.8 (0.4) | –0.3 (–1.2 to 0.5) | .44 |
| Unemotional | 0.1 (0.1) | 0.3 (0.2) | –0.2 (–0.7 to 0.3) | .51 |
| ***Parent measures*** | | | | |
| Parenting scale | | | | |
| Total | 0.6 (0.0) | 0.5 (0.0) | 0.0 (–0.1 to 0.2) | .49 |
| Laxness | 0.4 (0.0) | 0.4 (0.1) | –0.0 (–0.2 to 0.2) | .98 |
| Overreactivity | 0.8 (0.1) | 0.7 (0.1) | 0.1 (–0.1 to 0.3) | .16 |
| Hostility | 0.3 (0.0) | 0.3 (0.1) | 0.0 (–0.1 to 0.2) | .60 |
| DASS-21^e^ | | | | |
| Total | 6.1 (0.7) | 3.3 (1.2) | 2.8 (0.0 to 5.6) | .049 |
| Depression | 2.0 (0.3) | 0.6 (0.5) | 1.4 (0.2 to 2.6) | .019 |
| Anxiety | 2.0 (0.2) | 0.4 (0.3) | 0.5 (–0.2 to 1.3) | .17 |
| Stress | 3.2 (0.4) | 2.3 (0.6) | 0.9 (–0.6 to 2.3) | .25 |

*Note*: ADHD = attention-deficit/hyperactivity disorder; ODD = oppositional defiant disorder; PDD = pervasive developmental disorder

^a^ Least-squares mean; ^b^Adjusted with maternal education and duration of problems; ^c^CBCL = Child Behavior Checklist; ^d^ICU= Inventory of Callous-Unemotional Traits; ^e^DASS-21= Depression Anxiety and Stress Scale Short Form, SE=standard error
