# Supplementary material for: Effectiveness of an Internet-Based and Telephone-Assisted Training for Parents of 4-Year-Old Children With Disruptive Behavior: Implementation Research
Source: J Med Internet Res. 2022 Apr 4;24(4):e27900. doi: 10.2196/27900 (PMC9016503; doi:10.2196/27900)
Supplement: Multimedia Appendix 4 [file jmir_v24i4e27900_app4.docx]

**Table S3.** Mean changes from baseline to 6 months in child psychopathology, parenting skills and parents’ stress in the Implementation and Randomized controlled trial (RCT) educational control groups.

| Variable | Mean^a^ (SE) Change From Baseline to 6 Months | | Implementation vs. RCT education control  Mean (95% CI) | *P* ^b^ value |
| --- | --- | --- | --- | --- |
|  | Implementation Mean^a^ (SE)  n=600 | RCT education control  Mean (SE)  n=232 |  |  |
| ***Child measures*** | | | | |
| **Primary outcome** | | | | |
| CBCL^c^ externalizing | 6.1 (0.4) | 3.8 (0.6) | 2.4 (0.9 to 3.9) | .002 |
| **Secondary outcomes** | | | | |
| CBCL^c^ Total | 14.7 (1.1) | 8.6 (1.7) | 6.1 (2.1 to 10.0) | .003 |
| CBCL^c^ Internalizing | 3.4 (0.4) | 2.0 (0.6) | 1.4 (0.0 to 2.8) | .046 |
| Symptom domains | | | | |
| Aggression | 5.4 (0.4) | 3.3 (0.6) | 2.1 (0.8 to 3.4) | .002 |
| Attention | 0.7 (0.1) | 0.4 (0.1) | 0.3 (–0.1 to 0.6) | .28 |
| Sleep | 1.5 (0.1) | 0.8 (0.2) | 0.6 (0.1 to 1.2) | .016 |
| Withdrawn | 0.7 (0.5) | 0.2 (0.2) | 0.5 (0.1 to 0.9) | .007 |
| Somatic | 0.8 (0.1) | 0.6 (0.2) | 0.2 (–0.3 to 0.6) | .48 |
| Anxious | 0.8 (0.1) | 0.5 (0.2) | 0.4 (–0.1 to 0.8) | .08 |
| Emotional | 1.1 (0.1) | 0.8 (0.2) | 0.4 (–0.2 to 0.9) | .16 |
| DSM-5 subscores | | | | |
| Affective problems | 1.2 (0.1) | 0.7 (0.2) | 0.5 (0.1 to 1.0) | .021 |
| Anxiety problems | 1.3 (0.1) | 0.7 (0.2) | 0.6 (0.1 to 1.1) | .027 |
| PDD problems | 1.3 (0.2) | 0.6 (0.3) | 0.7 (0.1 to 1.3) | .029 |
| ADHD problems | 1.5 (0.1) | 0.9 (0.2) | 0.6 (0.1 to 1.1) | .026 |
| ODD problems | 1.9 (0.1) | 1.2 (0.2) | 0.6 (0.2 to 1.1) | .010 |
| Parenting scale | | | | |
| Total | 0.6 (0.0) | 0.1 (0.0) | 0.5 (0.4 to 0.6) | <.001 |
| Laxness | 0.4 (0.0) | 0.1 (0.1) | 0.4 (0.2 to 0.5) | <.001 |
| Overreactivity | 0.8 (0.1) | 0.1 (0.1) | 0.7 (0.5 to 0.8) | <.001 |
| Hostility | 0.3 (0.0) | 0.0 (0.1) | 0.3 (0.1 to 0.4) | .001 |
| ICU^d^ | | | | |
| Total | 3.9 (0.4) | 3.0 (0.7) | 0.9 (–0.7 to 2.4) | .27 |
| Callousness | 2.1 (0.2) | 1.1 (0.3) | 1.0 (0.2 to 1.7) | .012 |
| Uncaring | 1.6 (0.2) | 1.7 (0.3) | –0.1 (–0.9 to 0.6) | .73 |
| Unemotional | 0.1 (0.1) | 0.1 (0.2) | 0.0 (–0.4 to 0.5) | .91 |
| ***Parent measures*** | | | | |
| Parenting scale | | | | |
| Total | 0.6 (0.0) | 0.1 (0.0) | 0.5 (0.4 to 0.6) | <.001 |
| Laxness | 0.4 (0.0) | 0.1 (0.1) | 0.4 (0.2 to 0.5) | <.001 |
| Overreactivity | 0.8 (0.1) | 0.1 (0.1) | 0.7 (0.5 to 0.8) | <.001 |
| Hostility | 0.3 (0.0) | 0.0 (0.1) | 0.3 (0.1 to 0.4) | .001 |
| DASS^e^ | | | | |
| Total | 6.1 (0.7) | 2.1 (1.1) | 4.1 (1.4 to 6.7) | .003 |
| Depression | 2.0 (0.3) | 0.8 (0.5) | 1.3 (0.1 to 2.4) | .029 |
| Anxiety | 0.9 (0.2) | –0.0 (0.3) | 1.0 (0.2 to 1.7) | .014 |
| Stress | 3.2 (0.4) | 1.9 (0.7) | 1.9 (0.6 to 3.2) | .005 |

*Note*: ADHD = attention-deficit/hyperactivity disorder; ODD = oppositional defiant disorder; PDD = pervasive developmental disorder

^a^Least-squares means; ^b^Adjusted with maternal education, duration of problems and paternal age.; ^c^CBCL = Child Behavior Checklist.; ^d^ICU= Inventory of Callous-Unemotional Traits.; ^e^DASS-21= Depression Anxiety and Stress Scale Short Form, SE = standard error.
