# Supplementary material for: Effectiveness of an Internet-Based and Telephone-Assisted Training for Parents of 4-Year-Old Children With Disruptive Behavior: Implementation Research
Source: J Med Internet Res. 2022 Apr 4;24(4):e27900. doi: 10.2196/27900 (PMC9016503; doi:10.2196/27900)
Supplement: Multimedia Appendix 5 [file jmir_v24i4e27900_app5.docx]

**Table S4**. Change from baseline to 6 months in child psychopathology, parenting skills and parents’ stress in the implementation and RCT intervention groups. The city of Turku is excluded from the implementation data.

| Variable | Mean (SE) change from baseline to 6 months | | Implementation versus RCT  mean (95% CI) | *P*^b^ value |
| --- | --- | --- | --- | --- |
|  | Implementation  mean^a^ (SE)  n=514 | RCT  mean^a^ (SE)  n=176 |  |  |
| ***Child measures*** | | | | |
| **Primary outcome** | | | | |
| CBCL^c^ Externalizing | 6.1 (0.4) | 6.1 (0.6) | –0.0 (–1.5 to 1.5) | .96 |
| **Secondary outcomes** | | | | |
| CBCL^c^ Total | 14.8 (1.1) | 14.7 (1.7) | 0.2 (–3.8 to 4.1) | .94 |
| CBCL^c^ Internalizing | 3.4 (0.4) | 3.4 (0.6) | 0.1 (–1.3 to 1.4) | .93 |
| Symptom domains | | | | |
| Aggression | 5.4 (0.4) | 5.5 (0.5) | –0.1 (–1.4 to 1.2) | .83 |
| Attention | 0.7 (0.1) | 0.6 (0.1) | 0.1 (–0.2 to 0.5) | .55 |
| Sleep | 1.5 (0.2) | 1.5 (0.2) | –0.0 (–0.6 to 0.5) | .88 |
| Withdrawn | 0.7 (0.1) | 0.5 (0.2) | 0.2 (–0.1 to 0.6) | .25 |
| Somatic | 0.8 (0.1) | 0.6 (0.2) | 0.2 (–0.2 to 0.7) | .36 |
| Anxious | 0.8 (0.1) | 1.0 (0.2) | –0.2 (–0.6 to 0.3) | .44 |
| Emotional | 1.1 (0.2) | 1.3 (0.2) | –0.2 (–0.7 to 0.3) | .46 |
| DSM-5 subscores | | | | |
| Affective problems | 1.2 (0.1) | 1.3 (0.2) | –0.1 (–0.5 to 0.4) | .79 |
| Anxiety problems | 1.3 (0.2) | 1.5 (0.2) | –0.2 (–0.8 to 0.3) | .41 |
| PDD problems | 1.3 (0.2) | 1.2 (0.3) | 0.2 (–0.5 to 0.8) | .61 |
| ADHD problems | 1.6 (0.2) | 1.2 (0.2) | 0.3 (–0.2 to 0.9) | .20 |
| ODD problems | 1.8 (0.1) | 2.2 (0.2) | –0.4 (–0.9 to 0.1) | .10 |
| ICU^d^ |  |  |  |  |
| Total | 5.0 (0.5) | 4.5 (0.5) | 0.5 (–0.9 to 2.0) | .44 |
| Callousness | 2.6 (0.2) | 2.1 (0.3) | 0.5 (–0.2 to 1.2) | .16 |
| Uncaring | 2.3 (0.2) | 2.0 (0.3) | 0.4 (–0.3 to 1.1) | .29 |
| Unemotional | 0.1 (0.1) | 0.4 (0.2) | –0.3 (–0.8 to 0.1) | .13 |
| ***Parent measures*** | | | | |
| Parenting scale | | | | |
| Total | 0.6 (0.0) | 0.5 (0.0) | 0.0 (–0.1 to 0.1) | .65 |
| Laxness | 0.4 (0.0) | 0.4 (0.1) | 0.0 (–0.2 to 0.2) | .91 |
| Overreactivity | 0.8 (0.1) | 0.6 (0.1) | 0.1 (–0.0 to 0.3) | .13 |
| Hostility | 0.3 (0.0) | 0.3 (0.1) | –0.0 (–0.2 to 0.1) | .83 |
| DASS^e^ | | | | |
| Total | 6.0 (0.8) | 3.9 (1.1) | 2.1 (–0.6 to 4.9) | .12 |
| Depression | 2.0 (0.3) | 1.0 (0.5) | 1.0 (–0.1 to 2.1) | .07 |
| Anxiety | 1.0 (0.2) | 0.8 (0.3) | 0.2 (–0.5 to 0.9) | .57 |
| Stress | 3.1 (0.4) | 2.2 (0.6) | 0.9 (–0.5 to 2.3) | .20 |

*Note*: ADHD = attention-deficit/hyperactivity disorder; ODD = oppositional defiant disorder; PDD = pervasive developmental disorder.^a^Least-squares mean ^b^Adjusted with maternal education and duration of problems. ^c^CBCL = Child Behavior Checklist. ^d^ ICU= Inventory of Callous-Unemotional Traits. ^e^DASS-21= Depression Anxiety and Stress Scale Short Form. SE=standard error.
