# Supplementary material for: Effectiveness of an Internet-Based and Telephone-Assisted Training for Parents of 4-Year-Old Children With Disruptive Behavior: Implementation Research
Source: J Med Internet Res. 2022 Apr 4;24(4):e27900. doi: 10.2196/27900 (PMC9016503; doi:10.2196/27900)
Supplement: Multimedia Appendix 6 [file jmir_v24i4e27900_app6.docx]

**Table S5**. Change from baseline to post-treatment and 6 months in daily activities and social interactions in the Implementation group (n=600).

| Variable | Baseline to post-treatment | | Baseline to 6 months | | Post-treatment to 6 months | |
| --- | --- | --- | --- | --- | --- | --- |
|  | Mean  (95% Cl) | *P* ^b^ value | Mean^a^  (95% Cl) | *P* ^b^ value | Mean^a^  (95% Cl) | *P* ^b^ value |
| **Daily activities** | | | | | | |
| Getting dressed | 0.5 (0.5 to 0.6) | <.001 | 0.5 (0.4 to 0.6) | <.001 | –0.0 (–0.1 to 0.1) | .83 |
| Getting ready daycare | 0.4 (0.4 to 0.5) | <.001 | 0.5 (0.4 to 0.6) | <.001 | 0.1 (–0.0 to 0.2) | .22 |
| Dining behaviour | 0.4 (0.3 to 0.4) | <.001 | 0.5 (0.4 to 0.6) | <.001 | 0.1 (0.0 to 0.2 | .042 |
| Getting ready for bed | 0.5 (0.4 to 0.6) | <.001 | 0.6 (0.5 to 0.7) | <.001 | 0.2 (0.1 to 0.3) | .003 |
| **Social interactions** | | | | | | |
| Playing with other children | 0.3 (0.2 to 0.4) | <.001 | 0.2 (0.1 to 0.3) | <.001 | –0.1 (–0.2 to 0.0) | .08 |
| Playing with siblings | 0.3 (0.2 to 0.4) | <.001 | 0.4 (0.3 to 0.5) | <.001 | 0.1 (–0.1 to 0.2) | .39 |
| During car/bike ride | 0.1 (0.0 to 0.2) | .005 | 0.1 (0.0 to 0.2) | .013 | –0.0 (–0.1 to 0.1) | .88 |
| In public places | 0.5 (0.4 to 0.6) | <.001 | 0.5 (0.4 to 0.6) | <.001 | 0.0 (–0.1 to 0.2) | .62 |

^a^Least-squares means

^b^Adjusted with maternal education and duration of problems.
